# Quantum Topological Neuristors for Advanced Neuromorphic Intelligent Systems

**DOI:** 10.1002/advs.202300791

**Published:** 2023-06-21

**Authors:** Dani S. Assi, Hongli Huang, Vaithinathan Karthikeyan, Vaskuri C. S. Theja, Maria Merlyne de Souza, Ning Xi, Wen Jung Li, Vellaisamy A. L. Roy

**Affiliations:** ^1^ Electronics and Nanoscale Engineering James Watt School of Engineering University of Glasgow Glasgow G12 8QQ UK; ^2^ Materials Science and Engineering City University of Hong Kong Tat Chee Avenue Hong Kong Hong Kong; ^3^ Electronics and Electrical Engineering The University of Sheffield Sheffield S3 7HQ UK; ^4^ Industrial and Manufacturing Systems Engineering The University of Hong Kong Pokfulam Road Hong Kong Hong Kong; ^5^ Mechanical Engineering City University of Hong Kong Tat Chee Avenue Hong Kong Hong Kong; ^6^ School of Science and Technology Hong Kong Metropolitan University Ho Man Tin Hong Kong Hong Kong

**Keywords:** artificial neural network, artificial synapse, intelligent systems, neuromorphic devices, neuromorphic perception, synaptic device, topological insulator

## Abstract

Neuromorphic artificial intelligence systems are the future of ultrahigh performance computing clusters to overcome complex scientific and economical challenges. Despite their importance, the advancement in quantum neuromorphic systems is slow without specific device design. To elucidate biomimicking mammalian brain synapses, a new class of quantum topological neuristors (QTN) with ultralow energy consumption (pJ) and higher switching speed (µs) is introduced. Bioinspired neural network characteristics of QTNs are the effects of edge state transport and tunable energy gap in the quantum topological insulator (QTI) materials. With augmented device and QTI material design, top notch neuromorphic behavior with effective learning‐relearning‐forgetting stages is demonstrated. Critically, to emulate the real‐time neuromorphic efficiency, training of the QTNs is demonstrated with simple hand gesture game by interfacing them with artificial neural networks to perform decision‐making operations. Strategically, the QTNs prove the possession of incomparable potential to realize next‐gen neuromorphic computing for the development of intelligent machines and humanoids.

## Graphical abstract

This work illustrates a novel class of quantum topological neuristors specifically developed for the neuromorphic systems, characterized by ultralow switching energy to biomimic mammalian brain synapses. Top‐notch bioinspired performance and efficient neural network characteristics of neuristors are incurred from the unique edge state transport properties and tunable energy gap of quantum topological insulator materials.

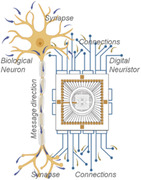

## 1 Introduction

Synapses are the basic processing units in human brain responsible for daily operations and memory.^[^ ^1^, ^2^ ^]^ With 10^15^ synapse interconnecting 10^11^ neurons and only 10 femtojoules/synaptic process, the brain itself requires less than one‐millionth of the power consumed by a supercomputer for a process.^[^ ^3^, ^4^, ^5^ ^]^ In the fact, over 8% of consumed global electricity are for computing devices which is also doubling in every decade, there is a challenging desire for developing efficient design of new materials and devices with low energy requirements compared to the conventional Si‐cMOS based architecture.^[^ ^6^, ^7^, ^8^, ^9^ ^]^ In this direction, by biomimicking the human nervous system and their biological synapse units, great efforts are underway to produce an artificial neuromorphic synapse device to learn and respond to the electronic neural signals from other artificial sensory devices/systems.^[^ ^10^, ^11^, ^12^, ^13^ ^]^

### 1.1 Conventional Design of Neuromorphic Electronics

Traditionally, silicon‐based complementary metal–oxide semiconductor are used for the design of neuromorphic computing systems to mimic the human biological neural systems for energy efficient operations.^[^ ^6^ ^]^ Recently, Si‐cMOS‐based prototypes for neuromorphic chips were demonstrated in major projects such as SpiNNaker,^[^ ^14^ ^]^ TrueNorth,^[^ ^15^ ^]^ NeuroGrid,^[^ ^16^ ^]^ BrainScaleS,^[^ ^17^ ^]^ and Braindrop.^[^ ^18^ ^]^ Though these projects demonstrate a level of energy efficiency, they are limited in their dynamic scalability, device volatility and ineffective plasticity affects their applications in adaptivity learning and complex processes.^[^ ^19^, ^20^ ^]^ Also, the traditional silicon cMOS architecture has reached the limit of key quantities like Size, Weight and Power (SWaP) for further scalability in device performance. This arises an additional challenge to reduce SWaP in artificial synaptic device with augmented device architecture.^[^ ^21^ ^]^ Advantageously toward artificial synapse, memristors possess a great advantage to satisfy the SWaP requirements of excellent scalability, nonvolatile devices integrated with logics and memory units.^[^ ^22^, ^23^, ^24^ ^]^

### 1.2 Postsilicon Neuromorphic Electronics

On the contrary to cMOS devices, memristors are bioinspired electronic devices with exact functions of biological synaptic and neural systems.^[^ ^25^, ^26^ ^]^ Unlike in cMOS devices, the basic device architecture of memristors is relatively simple with an active layer material of choice between two electrodes. Concurrently, this device design also resembles the biological synapses interconnecting pre/postsynaptic neuron clusters.^[^ ^20^, ^27^ ^]^ With organic/inorganic and oxide‐based semiconductors as active materials in memristor design, various underlying mechanisms like filament formation,^[^ ^28^ ^]^ ionic conduction,^[^ ^29^ ^]^ trapping/detrapping,^[^ ^30^ ^]^ defect migration,^[^ ^31^ ^]^ and redox reactions^[^ ^32^ ^]^ are being put forth as efficient synaptic switching process prove their neuromorphic functions.^[^ ^24^ ^]^ Based on the material of fabrication, their characteristic response to an applied external (optical or electric or magnetic) stimulus varies owing to various factors such as energy consumption, electronic conduction, and phase fluctuations.^[^ ^33^ ^]^ Despite the effective synaptic device design and mechanisms, the feasibility of training the memristive synaptic device to respond to external stimuli with ultralow power consumption (pJ) is poor, as they are specifically designed for memory and logic gates. Thus, it is well understood that the long‐standing issue with development of neuromorphic devices/systems for commercialization is the lack of efficient materials to mimic biological synapses with equivalent performance as the brain.^[^ ^34^, ^35^, ^36^, ^37^, ^38^ ^]^

### 1.3 Quantum Topological Insulators

In this regard, quantum topological insulators (QTI) with topological phases illustrates a unique edge conducting states arising from their time reversal invariant band inversion.^[^ ^21^, ^39^ ^]^ These properties make them resistant to defect induced back scattering and spin–momentum locking at the surface states creates a topologically nontrivial narrow energy gap at room temperature.^[^ ^40^ ^]^ The proven nontrivial electronic characteristics of topological insulator materials are distinct from metals and insulators, making them a special class of quantum materials.^[^ ^41^ ^]^ The edge state transport with tunable energy gap in topological insulator materials makes them the perfect candidate for implementing artificial synaptic device for neuromorphic systems, as the synaptic switching current for turning of the device from nonconducting to conducting state flows via the edge states. Ultimately, the edge state transport consumes ultralow energy with high switching speed.^[^ ^39^, ^42^ ^]^ Most recently, QTIs like Bi_2_Se_3_ and Bi_2_Te_3_ are being explored deeply for their application in low power electronic devices and has proved to be most efficient in comparison to conventional silicon devices.^[^ ^41^, ^43^, ^44^, ^45^, ^46^, ^47^, ^48^, ^49^ ^]^

### 1.4 Quantum Topological Neuristors Design

**Figure 1:**
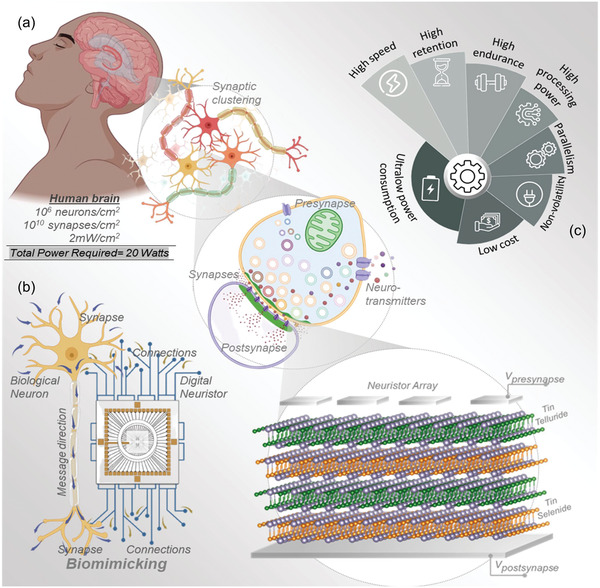
Biomimicking hierarchy from human brain to quantum topological neuristor. a) Conceptual illustration of the quantum topological neuristor design derivation from the synaptic neuron clusters function in human brain. b) Physical characteristics and communication pathway comparison of biological neuron to the artificial synaptic neuristor array. c) Advantages of quantum topological insulator materials for realizing artificial synaptic neuromorphic functions.

In the stage of developing a quantum neuromorphic supercomputer with equivalent power requirements as human brain, the primary requirement of realization is to fabricate an effective artificial synaptic device unit.^[^ ^50^ ^]^ By hybridizing QTI materials with memristive device design, here we introduce development of a new class of artificial synaptic device, neuristors, explicitly for neuromorphic systems. We use optimized proportions of 2D layered tin selenide (SnSe) and tin telluride (SnTe) QTIs via electrochemical process for the growth of synaptic clusters. **Figure** **1**a conceptually illustrates the hierarchical order in the bioinspired development of QTI‐based artificial synaptic neuristors. Here, our QTI‐based artificial synaptic neuristor design is an exact replica of biological synapses with postsynaptic and presynaptic electrode array/connections as silver contacts, neurotransmitters as trapped charge carriers. The conceptual Figure 1a explains the emulation process of QTI‐based artificial synaptic device from the biological synaptic cluster formations. Figure 1b depicts the structural resemblance of a neuron to our developed array of synaptic neuristors. Moreover, the communication signal pathways are more homogenously biomimicked between the synaptic dendrites in neurons and the artificial synaptic neuristor array. Figure 1c shows the material compatible advantages of using topological insulator materials for developing artificial synaptic neuristors. Optimizing the interplay of quantum mechanics in the topologically protected surface states of SnSe_1−_ *_x_* Te*_x_* will facilitate to achieve massive parallelism, high endurance and scalability in our quantum topological neuristors. With this approach, we meticulously emulate the biological synaptic actions in quantum topological neuristors (QTN) demonstrating high switching speed (µs) and ultralow power consumption (pJ). Moreover, we exhibit the neuromorphic potentials of our QTNs by their short‐/long‐term memory capacity with learning and forgetting cycle characteristics. The effective neuromorphic performance of the QTNs is also illustrated by associating with artificial neural networks (ANNs) through pattern recognition of hand gestures. With these first‐rate neuromorphic characteristics, QTNs prove to be the first of kind artificial synaptic device with incomparable potential for next generation artificial intelligence like prosthetics and humanoids.

## 2 Results

### 2.1 QTI Material Properties

**Figure 2:**
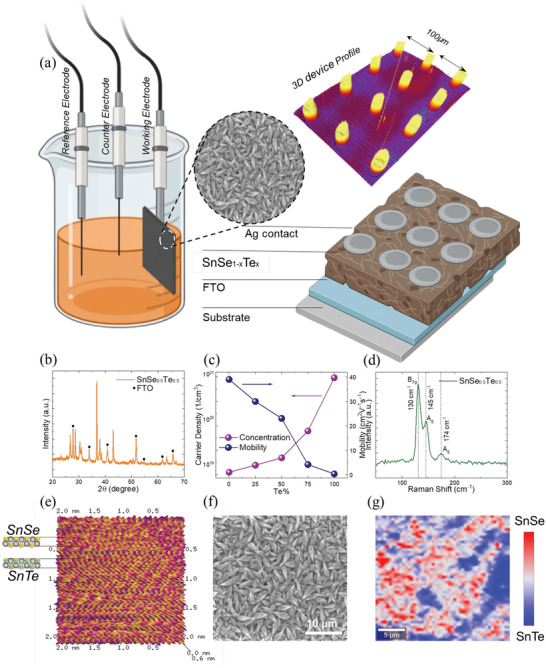
Material and experimental characterization of the QTNs. a) Representation of the three‐electrode electrochemical deposition (ECD) method to fabricate the QTNs. b) XRD analysis of the SnSe_0.5_Te_0.5_ thin film. c) Charge carrier dynamics in QTNs for different Te concentration. d) Raman pattern of the SnSe_0.5_Te_0.5_ QTN. e) AFM representation of layer‐by‐layer formation of SnSe and SnTe. f) FESEM image illustration of a uniform growth of grain‐shaped leaves of the SnSe_0.5_Te_0.5_ thin film. g) Raman mapping representation of homogenous distribution of SnSe_0.5_Te_0.5_ thin film growth.

QTNs are designed using QTI materials and are fabricated using electrochemical process with thin film proportions of SnSe*_x_* Te_1−_ *_x_* (*x* = 0, 0.25, 0.50, 0.75, and 1). Structurally, thin film QTNs are designed with the conductive oxide film as presynaptic neuron and silver electrodes as postsynaptic neuron to form a synaptic cleft with topological edge state transport of charge carriers as neurotransmitters. Schematic of the neuristor structure and the experimental setup for fabrication are as illustrated in **Figure** **2**a. To characterize the fabricated QTI materials, we performed X‐ray diffraction analysis as shown in Figure 2b which confirms the presence of SnSe_0.5_Te_0.5_ phase (JCPDS No. 48‐1224) with additional peaks of FTO (JCPDS No. 41‐1445) substrate indicating the uniformity of QTI materials deposited. Crystalline phase changes with respect to the proportion of SnSe_1−_ *_x_* Te*_x_* are compared through the X‐ray diffraction pattern shown in Figure S1 (Supporting Information). In order to understand thin film quality, atomic force microscopy was performed where the presence of layer‐by‐layer formation of SnSe and SnTe is observed. This layer‐by‐layer presence of Sn—Se—Te is confirmed by the *d*‐spacing corresponding to SnSe (*d* = 0.35 nm) and SnTe (*d* = 0.317 nm) as demonstrated in Figure 2e. Field emission scanning electron microscopy (FESEM) images of thin films illustrate the presence of uniform growth of leaves like grains as shown in Figure 2f, whose EDX plot shown in Section S2 (Supporting Information) also proves the uniformity in SnSeTe composition and elemental distribution. Further to visually demonstrate the proportion of QTI layers, we performed the Raman mapping analysis as shown in Figure 2g, from the Raman data the peaks 130, 145, and 174 cm^−1^ correspond to B_3g_ and A_g_ Raman vibration modes of SnSe_0.5_Te_0.5_ phase. The relation between the QTI proportions and the electrical conductivity was analyzed using room‐temperature Hall measurement system, where the carrier concentration increases with increase in the Te proportions. The obtained charge carrier transport results match the change in morphology and QTI film growth.

### 2.2 Quantum Topological Neuristor Characteristics

QTNs IV switching characteristics between the pre‐ and postsynaptic neurons for varied proportion of SnSe_1−_ *_x_* Te*_x_* (*x* = 0, 0.25, 0.50, 0.75, and 1) were performed. The different synaptic switching mechanisms involved in the transition from high resistance state (HRS) to low resistance state (LRS) for varied proportion of SnSe_1−_ *_x_* Te*_x_* are demonstrated in Figures S4–S8 (Supporting Information). Different mechanisms involved in our switching process are thermionic emission limited conduction, ohmic conduction, space charge limited current, and trap charge limited current (TCLC). As shown in Figure S4b (Supporting Information) in region A, overcoming the energy barrier, excitation of charges into the active layer by thermal activation are witnessed following the relation in Equation (1). Region B exhibits a linear voltage–current relationship attributing to the presence of ohmic conduction model following Equation (2). Following the relation (3), region C demonstrates the characteristics of the space charge limited current attributed from accumulation of injected charge carriers in the active layers of SnSe_1−_ *_x_* Te*_x_* between the electrodes forming a capacitance model. Consecutively according to Child's law, owing to the accumulation of charge trapping in the active layer causes change in the synaptic resistance state of the neuristor by switching from HRS to LRS following trapped charge limited current model.^[^ ^51^ ^]^

(1) $$I\propto A*T^{2}\exp\left[-\frac{q\phi_{0}}{kT}+q{\left(\frac{q^{3}V}{4\pi \varepsilon }\right)}^{\frac{1}{2}}\right]$$

(2) $$I\propto Vexp\frac{\left(-\Delta E_{e}\right)}{kT}$$

(3) $$I\propto V^{\alpha }$$

**Figure 3:**
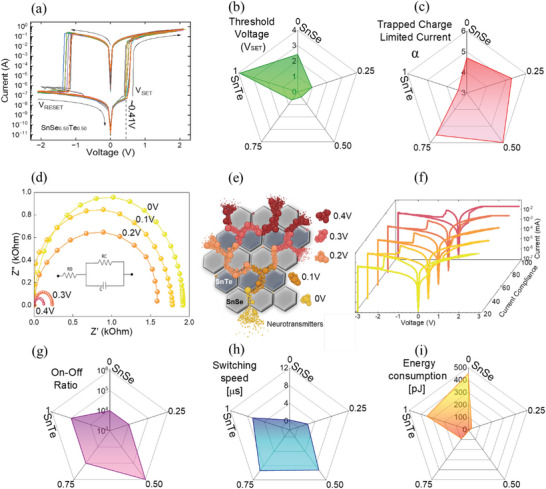
Electrical characterization of QTI neuristors. a) Demonstration of *I*–*V* characteristic of the SnSe_0.5_Te_0.5_ QTN with high repeatability. b) Average mean values of synaptic switching threshold voltage representation for varied proportion of SnSe_1−_ *_x_* Te*_x_* (*x* = 0, 0.25, 0.50, 0.75, and 1). c) Average mean values of trapped charged limited current (TCLC) levels for QTNs with different Te concentration. d) Impedance analysis under increasing voltage bias for the SnSe_0.5_Te_0.5_ QTN. e) Illustration represents the increase capacitance values with increase applied voltage for the trapping of charged in the QTI layers supported by the edge conduction states. f) Representation of the multilevel synaptic capability for the SnSe_0.5_Te_0.5_ QTN, proving their multifunctional operations. g) Average mean values of on/off ratio characteristics for QTNs with different Te concentration ranging from 0% to 100%. h) Average mean values of synaptic switching speed reliability in QTNs. i) Energy consumption comparison of QTNs with different Te concentration.

This trapped charge limited current model is the main phenomenon leading to transition between resistance states.^[^ ^51^ ^]^ The level of TCLC in the SnSe_1−_ *_x_* Te*_x_* neuristors can be estimated from the constant *α* as illustrated in **Figure** **3**c. Among the different Se/Te ratio, SnSe_0.5_Te_0.5_ demonstrates the best energy efficient ultralow threshold set voltage characteristics as shown in Figure 3a. This phenomenon of low switching voltage derives from the edge state transport properties and trap‐charge limit‐current constant (*α*) developed by charge trap states in of SnSe_1−_ *_x_* Te*_x_* QTIs. The average mean variation in *V*_SET_ and *α* with change in QTI proportions is illustrated in Figure 3b,c, as shown the sample with mixed proportions of QTIs demonstrates decrease in *V*_SET_ and increased *α*.

This effect is experienced from the increasing charge traps and the enhancement in edge conduction states in the interlaced layered structure of SnTe in SnSe matrix, whereas the pristine QTNs suffer from bulk conduction hence requires higher *V*_SET_ to perform the synaptic functions. To prove this phenomenon, we performed the impedance analysis under increasing voltage bias as shown in Figure 3d, which elucidates the changing resistance modes owing to the trapped charges in the interlaced quantum topological layers. The increase in capacitance values with the increasing voltage bias describes the trapping of charges in the QTI layers thereby supporting the edge conduction states for high‐speed synaptic switching process as shown in Table S3 (Supporting Information). The illustration for the potential change in conduction state with applied bias voltage is shown with the diagram in Figure 3e. From these outcomes, QTNs prove their characteristics of ultralow set threshold voltage to establish a synaptic process. In addition, Figure 3f shows the excellent capability of multilevel synaptic switching controlled by current compliance in QTNs proving their multifunctional parallel operations. Crucial component of any memory device possesses a larger on/off ratio, however, obtaining a larger on/off ratio with ultralow *V*_SET_ is challenging as it requires unique material properties. Strategically, the use of QTI for establishing a synaptic process makes them unique with distinctly voltage‐controlled edge state transport which supports a large on/off ratio of 10^6^ with a steady endurance over 10^5^ as demonstrated in Figure 3g,h.

As a result of the optimized material design in QTNs, higher energy efficiency in the synaptic process is achieved with a lowest energy consumption of ≈10 pJ for the proportion of SnSe_0.5_Te_0.5_ with a switching speed of ≈10 µs. A data‐driven set of comparison of energy consumption and switching speed for varied proportion of SnSe_1−_ *_x_* Te*_x_* is illustrated in Figures S9 and S10 (Supporting Information). Our results demonstrate that the synaptic switching performance in quantum topological insulators is exceptionally good when compared to the conventional organics, inorganics, and oxides‐based devices as shown in Tables S1 and S2 (Supporting Information). This potential of the proposed QTNs will make the future of neuromorphic systems energy efficient and more reliable for real‐time applications.

### 2.3 QTNs Neuromorphic Behavior

**Figure 4:**
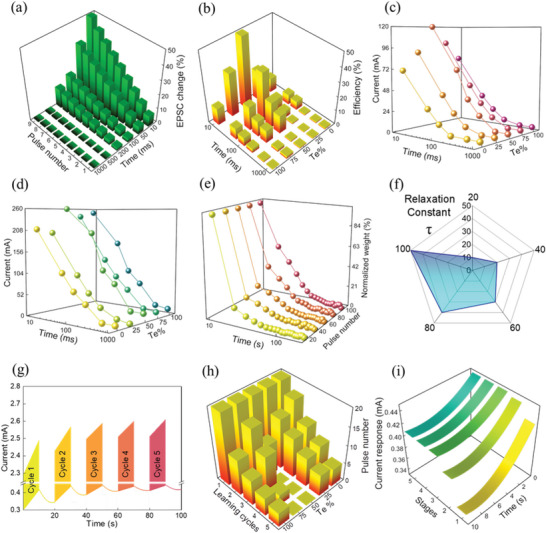
QTNs neuromorphic behavior of QTNs for varied proportion of SnSe_1−_ *_x_* Te*_x_* (*x* = 0, 0.25, 0.50, 0.75, and 1). a) Exhibitory postsynaptic current change of QTNs for applied electrical pulses with different interval time ranging from 10 ms to 1 s b) Plasticity efficiency. c) Paired pulse facilitation (PPF) synaptic function of QTNs. d) Post tetanic potentiation (PTP) synaptic function of QTNs. e) Transformation from short‐term potentiation (STP) to long‐term potentiation (LTP) with high efficiency in SnSe_0.5_Te_0.5_ QTN. f) Relaxation constant for the SnSe_0.5_Te_0.5_ QTN. g) Full learning‐relearning‐forgetting cycles for the SnSe_0.5_Te_0.5_ QTNs. h) Emulation of the learning and relearning cycles for the QTNs with different Te concentration. i) Decay‐induced forgetting stages for the SnSe_0.5_Te_0.5_ QTNs.

To prove the neuromorphic behavior of the developed QTNs, we study their effective learning, training, and memorizing properties using Hebbian synaptic plasticity (HSP) technique. HSP is a neural mechanism with ability to modify the synaptic weight via paired activation of presynaptic neuron with postsynaptic neuron.^[^ ^52^, ^53^, ^54^ ^]^ Hebbian synaptic learnings are crucial in mimicking process of biosynapses based on applied spike‐related computations, where spike time‐dependent plasticity (STDP) and spike rate‐dependent plasticity (SRDP) are two classic paradigms in mammalian brain for generation of the Hebbian synaptic plasticity, respectively. With respect to the brain cerebral behaviors, SRDP are entitled for transfer of data in biological neural networks which are directly connected to average action potential firing rate.^[^ ^55^, ^56^, ^57^ ^]^ To deeply investigate the SRDP learning process effectiveness and the effect of presynaptic spiking rate in QTNs, 10 consecutive spike pulses with a fixed amplitude of 200 mV and pulse width of 100 ms were applied to the synaptic neuristor device with the different pulse intervals as shown in **Figure** **4**a. The changing exhibitory postsynaptic device current (EPSC) with respect to the applied spike pulses is referred to as respective synaptic weight. With selected number of pulse trains, it is observed that shorter the intervals larger the synaptic response owing to the accumulation of applied charges in the QTNs. Successfully, this QTNs synaptic response mimics the biological synaptic response, thus proving the dependency of the presynaptic spiking rate to the strength of synaptic plasticity. To establish an ideal neuromorphic performance, we optimized the QTI material design with different proportions of interlaced SnTe in SnSe matrix, where the of SnSe_0.5_Te_0.5_ demonstrates maximum performance efficiency as shown in Figure 4b.

Biologically, synaptic communication between presynaptic and postsynaptic neurons occurs through the exchange of neurotransmitter molecules, induced by calcium (Ca^2+^) ions. When an action potential reaches the presynaptic axon terminal, Ca^2+^ channels open, allowing Ca^2+^ ions to rapidly enter the terminal. This influx of Ca^2+^ ions increase their cytoplasmic concentration, which diffuses across the synaptic cleft and selectively binds to receptors located on the membrane of the postsynaptic neuron. The resultant flow of calcium ions alters the conductance of the receiving neuron's membrane, which subsequently affects its membrane potential and can trigger an action potential.^[^ ^58^ ^]^ Analogous to the biological synaptic switching mechanism, in our artificial synaptic neuristors, upon applying an action potential to the presynaptic electrode the charge carriers as neurotransmitters are injected into the topological insulator electronic edge states and upon accumulated via the SCLC and TCLC mechanism it fires a triggering impulse to the postsynaptic electrode which marks a synaptic event. This process of enhancing the synaptic conduction, by amplifying the synaptic response for the second pulse is called the paired pulse facilitation function (PPF). Figure 4c demonstrates the imitation of this PPF neural facilitation which is the short‐term activity dependent form of synaptic plasticity.^[^ ^26^ ^]^ PPF characteristics of our developed QTNs are investigated by applying a pair of presynaptic pulses having different pulse intervals. As expected, when the paired pulse triggers the artificial synapse, the amplitude of the second pulse in postsynaptic neuron is much stronger compared to the first pulse. PPF synaptic function for different proportions of SnTe in SnSe was performed, for which the SnSe_0.5_Te_0.5_ demonstrates higher synaptic weight compared to other proportions.

Post‐tetanic potentiation (PTP) synaptic function is another short‐term dependent activity which also connects the short‐term plasticity (STP), learning and memory functions.^[^ ^59^ ^]^ PTP characteristics of our QTNs are investigated by sequential presynaptic pulse trains with varied intervals with the same measurement conditions as used in SRDP, and the results of their response are as shown in Figure 4d. Determined by their synaptic memory retention time, synaptic plasticity is categorized as STP and long‐term (LTP), where the LTP is majorly responsible for controlling neuronal plasticity, learning and memory operation, whereas STP for data processing, decoding information, and synaptic calculations. To evaluate those synaptic plasticity functions, the synaptic weights must be determined to classify the strength of the synaptic connections.^[^ ^60^, ^61^, ^62^, ^63^, ^64^, ^65^ ^]^ Figure 4e represents the normalized synaptic weight with respect to decay time with different number of pulse trains which shows the decrease in the decay time with increase in number of pulse trains as it changes from 0% to 45%. It is observed that the decay rate is significantly faster in the earlier pulse trains and much slower for the latter pulse trains, indicating the relaxation period of STP and LTP process, respectively. To fully investigate the transformation, form the STP to LTP, the exponential equation used as forgetting function in psychology was applied to analyze the decay process and to extract the relaxation time

(4) $$I=I_{0}+Aexp\left(-\frac{t}{\tau }\right)$$

where *I* is a weighted current at time *t*, *A* is a perfector of the equation, *τ* is relaxation constant measuring the decay rate, and *I*_0_ is a current constant.^[^ ^66^ ^]^ Figure 4f shows the relaxation constant *τ* versus number of applied pulse trains to the synaptic device. With increasing number of pulse trains, the relaxation constant *τ* also increases confirming successful transformation from STP to LTP in the SnSe_0.5_Te_0.5_ QTNs owing to repeated training process and learning operations.

Mammalian brain's empirical behaviors like learning, memorizing, and forgetting functions are adaptation of the biological neural network to the surrounding environment and day‐to‐day activities. Though they are not eternal, learning behavior is responsible mainly for getting the information and preserving previously acquired knowledge. Hence, the relearning process capacity are more significant for memory enhancement which simplifies the process of regaining previous forgotten knowledge.^[^ ^67^, ^68^, ^70^, ^71^, ^72^, ^73^ ^]^ Figure 4g represents the five cycles of 20 consecutive spike pulse trains applied to presynaptic neuron for SnSe_0.5_Te_0.5_ synaptic neuristors which emulates learning and relearning process represented by increments in conductance with applied synaptic pulse trains. When the application of pulse trains is stopped, the decay process begins, which is defined as forgetting process. In the learning and relearning cycles for the design optimized QTNs, the first cycle is defined as learning period consisting with the first pulse train applied to presynaptic neuron which is responsible for establishing the postsynaptic current (PSC) level for learning operation. The relearning stage begins from the second cycle and continues until the information gets fully memorized, which means that the entire cycle will have higher postsynaptic current level compared to first cycle. The efficiency of the learning‐relearning behavior stages of QTNs is directly connected to the amount of the relearning cycles necessary to fully cross postsynaptic current level established in learning period. From Figure 4h, we observed that the QTNs made with proportion of SnSe_0.5_Te_0.5_ are the most efficient and require only one relearning cycle to fully memorize the learning information. Efficiency increment is associated with the edge state transport and TCLC characteristics of QTNs. Figure 4i demonstrates the forgetting stages for SnSe_0.5_Te_0.5_ QTNs where the normalized current level is increasing with each cycle until its level is stabilized, proving that the information during the learning‐relearning operation got fully memorized.

### 2.4 QTI Sensory Neuromorphic System

Finally, to illustrate the capacity of our developed QTNs as a sensory neuromorphic system, we intended to create a hand gesture‐based gaming interface for proving a real‐time learning‐training‐forgetting performance efficiency. Need for development of effective hand gestures or sign languages recognition systems is in high demand as they are the only source of communication for persons with physical or mental impairment. Hence, the translation of gesture pattern to information via neuromorphic sensory systems will be more advantageous artificial intelligent technology.

**Figure 5:**
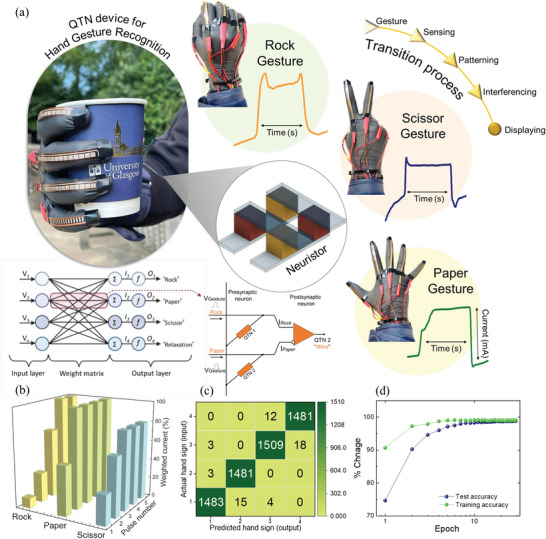
QTI sensory neuromorphic system. a) Demonstration of “Rock,” “Paper,” and “Scissor” hand gestures with their postsynaptic current pattern representation for each symbol. b) Training process of learning‐memorizing operation of QTN for each of the hand gesture. c) Confusion matrix between actual hand gesture input and the predicted hand gesture output. d) Comparison of the test and training accuracy with respect to our QTN neuromorphic system.

Here, we use flexible resistive sensor to train the QTNs with simple gaming gestures of “Rock,” “Paper,” and “Scissor” based on the change in current values with each gesture signs. The patterns of each gesture under different time intervals and stretching patterns were recorded for interfacing with ANN. Gesture translation and training process to the QTN sensory neuromorphic module was performed via the following steps: i) gesturing data collection, ii) sensing the gesture via QTN array, iii) patterning of signal response from QTNs, iv) interferencing the response signals and categorizing them via ANN, and v) displaying the translated output.^[^ ^74^ ^]^ In the construction of gesture recognition module incorporated with the QTNs, the flexible resistive sensors are connected in parallel to the QTN array with each device acting as a brain responding to the different training signals of hand gestures. To explore the QTN's performance under the real‐time conditions quantitatively, we created a simple single‐layer ANN with size of 4 × 3 and trained them with the gaming hand gesture of “Rock,” “Paper,” “Scissor,” and “Relax” patterns as shown in **Figure** **5**a. Though the present size of the single‐layer ANN is smaller, this prototype can be extended for large size of ANN with the flex resistive sensor array in the QTN array nodes. For the simulation process, we prepared over 10 000 trainings and 8000 gesture testing patterns consisting of four types of hand signs as shown in Figure 5a. Gesture training for QTNs was performed by applying the gesture pulses to be memorized and reproduced as shown in Figure 5b. The confusion matrix between actual hand sign input and the predicted hand sign output demonstrated in Figure 5c shows the ANN was trained after less than 100 training epochs. The training epoch shown in Figure 5d demonstrates the test and training accuracy with respect to percentage in training current. These demonstrations clearly prove the practicability of QTNs as an effective sensory neuromorphic system for hand gesture translation.

## 3 Conclusion

In this study, we successfully developed a quantum topological insulator based neuristor with equivalent neuromorphic synaptic performance as the mammalian brain. From our investigations, the quantum topological insulator materials are well exposed with their potential in advanced low power electronic switching systems. Surface state conduction in topological insulator materials facilitates liquid‐like charge flow between pre‐ and postsynaptic neuron layers representing the biological synaptic cleft operations. To optimize the material design, we investigated different proportions of 2D layered SnTe in SnSe matrix, thereby controlling the threshold set voltage in the neuristors. Furthermore, we prove the mechanism involved in low voltage switching is via the trapped charge limited current supported by the edge state transport in the QTI nanograin structure. Also, our QTNs demonstrate an ultrastable multistage synaptic switching capacity for multifunctionality, enabling high‐density data storage and processing behavior without sacrificing the physical dimensions of the device. Top notch, first‐rate neuromorphic characteristics of our proposed QTNs are successfully demonstrated by applying external presynaptic spike pulse trains. We represent the bionic modulations in QTNs via the fluctuations in the synaptic weight, representing the simulation of human neural network functions, including PPF and PTP synaptic function with learning‐relearning‐forgetting cycle effectiveness. Here, we deliberately claim that the energy requirement for the QTNs to perform the synaptic functions responding to external stimuli is in the range of 10 pJ, the lowest for any reported neuromorphic synaptic devices and which is 90 times lower than conventional Si‐cMOS neuromorphic circuits.^[^ ^68^, ^75^ ^]^ Finally, to demonstrate outstanding bionic characteristics, we effectively developed a sensory‐neuromorphic concept that incorporates the ANNs through a synaptic human–computer interface based on human‐gesture recognition which demonstrates an exceptional feasibility and exceptional recognition rate of 99%. Thus, the proposed QTNs are the first‐of‐a‐kind artificial synaptic devices with incomparable potential to become the new modern standard for the bioinspired neuromorphic device for next‐generation artificial intelligence applications.

## 4 Experimental Section

### Fabrication of the QTNs

Thin film quantum topological neuristors were fabricated over FTO‐coated glass substrate which acts as postsynaptic neurons. The active layer of quantum topological insulator SnSe_1−_ *_x_* Te*_x_* (*x* = 0, 0.25, 0.50, 0.75, 1) was deposited using the electrochemical deposition (ECD) technique.^[^ ^76^ ^]^ Top electrodes made with 100 nm of silver acting as a presynaptic neuron were deposited by the thermal evaporation technique. The preparation process of layered Ag/SnSe_1−_ *_x_* Te*_x_*/FTO neuristor is schematically illustrated in Figure 2a. Prior to the deposition, the FTO‐coated glass substrates were patterned, sequentially cleaned with DI water, acetone, and ethanol for 30 min in ultrasonic bath. The presented SnSe_1−_ *_x_* Te*_x_* thin film was fabricated using the three‐electrode ECD method, carried out in the Metrohm Autolab PGSTAT302 instrument. The Ag/AgCl double junction reference electrode and high‐purity platinum counter electrode were used to monitor and control the electrochemical potential during the fabrication process. The FTO with the sheet resistance of ≈7 Ω sq^−1^ and an exposed area of 1 cm^2^ performed the role of the working electrode. The distance between the cathode and anode was set up at 0.5 cm for every deposition. The deposition bath contains 50 mL of deionized water (>18 MΩ), taken from a Purite (L300450). First, under stirring, NaOH pellets were added to the DI solution until a pH of 12 was achieved. Second, 2 × 10^−3^ m of EDTA was added to the mixture, changing the pH from 12 to 9. The solution was set up for string until the mixture became fully transparent. The EDTA presence in the solution was observed to increase the deposition bath lifespan and increase the adhesion rate of the deposited film into the FTO glass. Third, under stirring, 2 × 10^−3^ m of SnCl_2_·2H_2_O was added to the solution. After that, the solution was sonicated for 10 min until becoming fully transparent. Then the mixture was gently purged with purified nitrogen gas for 60 min to eliminate any dissolved oxygen. The used EDTA is responsible for forming an Sn‐EDTA complex, preventing the Sn^2+^ ions from hydrolysis in an aqueous solution. Fourth, under stirring, SeO_2_ and TeO_2_ were added to the solution. The mixture immediately becomes brownish red. Finally, H_2_SO_4_ was added to the mixture to adjust the pH to 2.5. All the thin films were deposited by maintaining the bath temperature at 55 °C. After deposition, the samples were washed with deionized water. Then, the samples were annealed in a nitrogen atmosphere at 300 °C for 3 h. The SnSe_1−_ *_x_* Te*_x_* thin films were uniform in appearance, gray in color, and had good adherent to the FTO substrate.

### Material Characterization of the QTNs

Structural properties of SnSe_1−_ *_x_* Te*_x_* thin film were verified for each sample using the powder X‐ray diffraction (XRD) Rigaku Miniflex diffractometer. Raman properties were confirmed by Horiba Labram HR Raman System with a 532 nm laser. Carrier density and mobility were measured using Lakeshore Hall measurement system at room temperature. The carrier concentration increases, and the mobility decreases with increasing tellurium concentration. The obtained charge carrier transport results match the change in morphology and film growth as Te% increases. To understand the surface roughness and atomic arrangement, atomic force microscope (AFM) Bruker Dimension HPI was used. The scan size was set up for 2 nm with a scan rate of 0.996 Hz. The result reveals layer‐by‐layer deposition of SnSe and SnTe. FESEM Hitachi SU8240 was used to verify the surface morphology.

### Synaptic Switching and Neuromorphic Characterization of the QTNs

The electrical characterization of the QTNs was conducted using a probe station equipped with the Agilent Technologies B1500A Semiconductor Device Analyzer, with data acquisition facilitated by a digital storage oscilloscope.

*Synaptic Switching*: The switching properties (*I*–*V* characteristics) were measured by applying the sequential sweep voltages thresholds to the QTNs devices.

*Paired‐Pulse Facilitation*: The determination of the PPF function involved the application of two consecutive train stimuli to the presynaptic neuron, with varying pulse intervals spanning from 10 ms to 1 s, while maintaining a consistent pulse width of 100 ms and a pulse amplitude of +200 mV. The PPF value was derived through the formula PPF = A2/A1, where A1 and A2 represent the PSC responses of the first and second pulse, respectively.

*Post‐Tetanic Potentiation*: To determine the PTP function, ten consecutive train stimuli were applied to the presynaptic neuron with varied pulse intervals ranging from 10 ms to 1 s, while maintaining a pulse width of 100 ms and a pulse amplitude of +200 mV. The PTP value was calculated using the formula PTP = A10/A1, where A1 and A10 represent the PSC responses of the first and tenth pulses, respectively.

*Transition Process from Short‐ to Long‐Term Potentiation*: To evaluate the capability of the synaptic device to transition from STP to LTP, a sequence of consecutive train stimuli, consisting of 20, 40, 60, 80, and 100 pulses, was administered to the presynaptic membrane, while maintaining a pulse interval of 100 ms and a pulse amplitude of 200 mV. The acquired results were fitted and analyzed using an exponential equation commonly employed as a forgetting function in psychology (*I* = *I*_0_ + A*e ^(−t/^ *^*τ*^* ^)^), where *I*_0_ represents a constant current, *A* represents a constant amplitude, and *τ* measures the relaxation constant, reflecting the rate of decay, which is the value of interest.

*Learning‐Forgetting‐Relearning Process*: The memorization and forgetting functions were determined by applying 100 training stimuli to the presynaptic neuron, distributed across five cycles, with each cycle consisting of 20 pulses. During the measurement, the pulse interval was fixed to 100 ms and the pulse amplitude was set at 200 mV. The initial cycle was designated as the memorization stage, whereas the subsequent cycles were considered as relearning stages. Following each cycle, a spontaneous relaxation decay “*τ*” occurred, commonly referred to as the forgetting stage.^[^ ^51^ ^]^

### Integration of the Wearable Hand Glove Sensory Neuromorphic System

The developed sensory neuromorphic glove system integrates five flexible resistive sensors, one for each finger, that are parallel‐connected and directly integrated with the developed QTN device. The QTN device was then connected to a computer and semiconductor analyzer for real‐time data processing using Matlab software.^[^ ^74^ ^]^

The sensory neuromorphic system was trained using a single‐layer perceptron (SLP) ANN model with a size of 4 × 3. During the training process, over 10 000 training patterns and 8000 gesture testing patterns were developed and implemented, encompassing four distinct hand sign categories: “Rock,” “Paper,” “Scissors,” and “Relax.”

### Statistical Analysis

The data processing and analysis was performed by Origin and Matlab software.

## Conflict of Interest

The authors declare no conflict of interest.

## Author Contributions

D.S.A. and V.K.: conceptualization, methodology, visualization, formal analysis, writing – original draft; H.H., and V.C.S.T.: software, resources, data curation; M.M.D.S., N.X., and W.J.L.: validation, investigation, writing – review & editing; and V.A.L.R.: conceptualization, supervision, funding acquisition, project administration, writing – review & editing.

## Supporting information

Supporting Information

## Data Availability

The data that support the findings of this study are available from the corresponding author upon reasonable request.
