# Supplementary material for: Quantum Topological Neuristors for Advanced Neuromorphic Intelligent Systems
Source: Adv Sci (Weinh). 2023 Jun 21;10(24):2300791. doi: 10.1002/advs.202300791 (PMC10460853; doi:10.1002/advs.202300791)
Supplement: Supplementary file 1 — Supporting Information [file ADVS-10-2300791-s001.pdf]

ADVANCED SCIENCE
Open Access

Supporting Information

for *Adv. Sci.*, DOI 10.1002/advs.202300791

Quantum Topological Neuristors for Advanced Neuromorphic Intelligent Systems

Dani S. Assi, Hongli Huang, Vaithinathan Karthikeyan, Vaskuri C. S. Theja, Maria Merlyne de Souza, Ning Xi, Wen Jung Li and Vellaisamy A. L. Roy**

Supplementary Materials for

Quantum Topological Neuristors for Advanced Neuromorphic Intelligent Systems

Dani S. Assi, Hongli Huang, Vaithinathan Karthikeyan*, Vaskuri C. S. Theja, Maria Merlyne de Souza, Ning Xi, Wen Jung Li, Vellaisamy A. L. Roy*

This PDF file includes:

Figs. S1 to S12
Tables S1 to S3
References

Figure S1

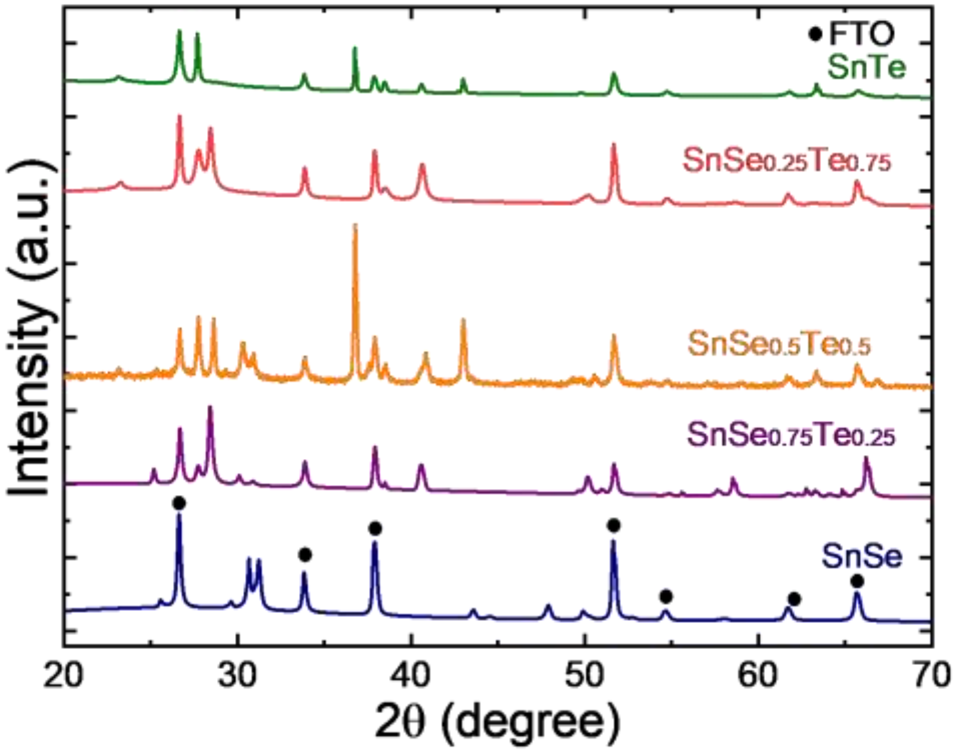

Figure S1: X-ray diffraction pattern for different proportions of $SnSe_{1-x}Te_x$ (x= 0, 0.25, 0.50, 0.75, 1).

Figure S2

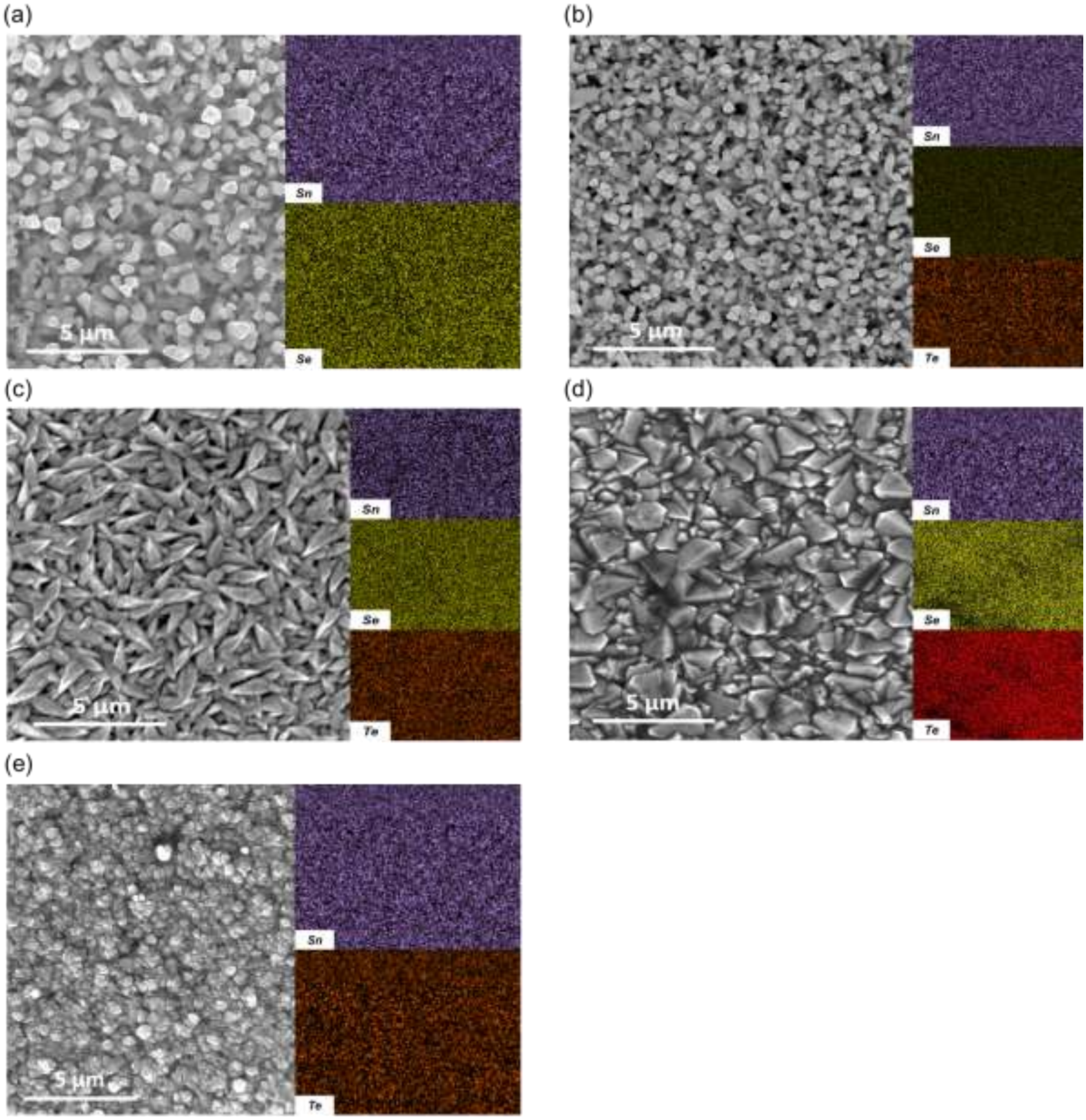

Figure S2: SEM images demonstrating the crystalline morphology change in (a) SnSe (b) $SnSe_{0.75}$Te0.$_{25}$ (c) $SnSe_{0.50}Te_{0.50}$ (d) $SnSe_{0.25}$Te0.$_{75}$ (e) SnTe.

Figure S3

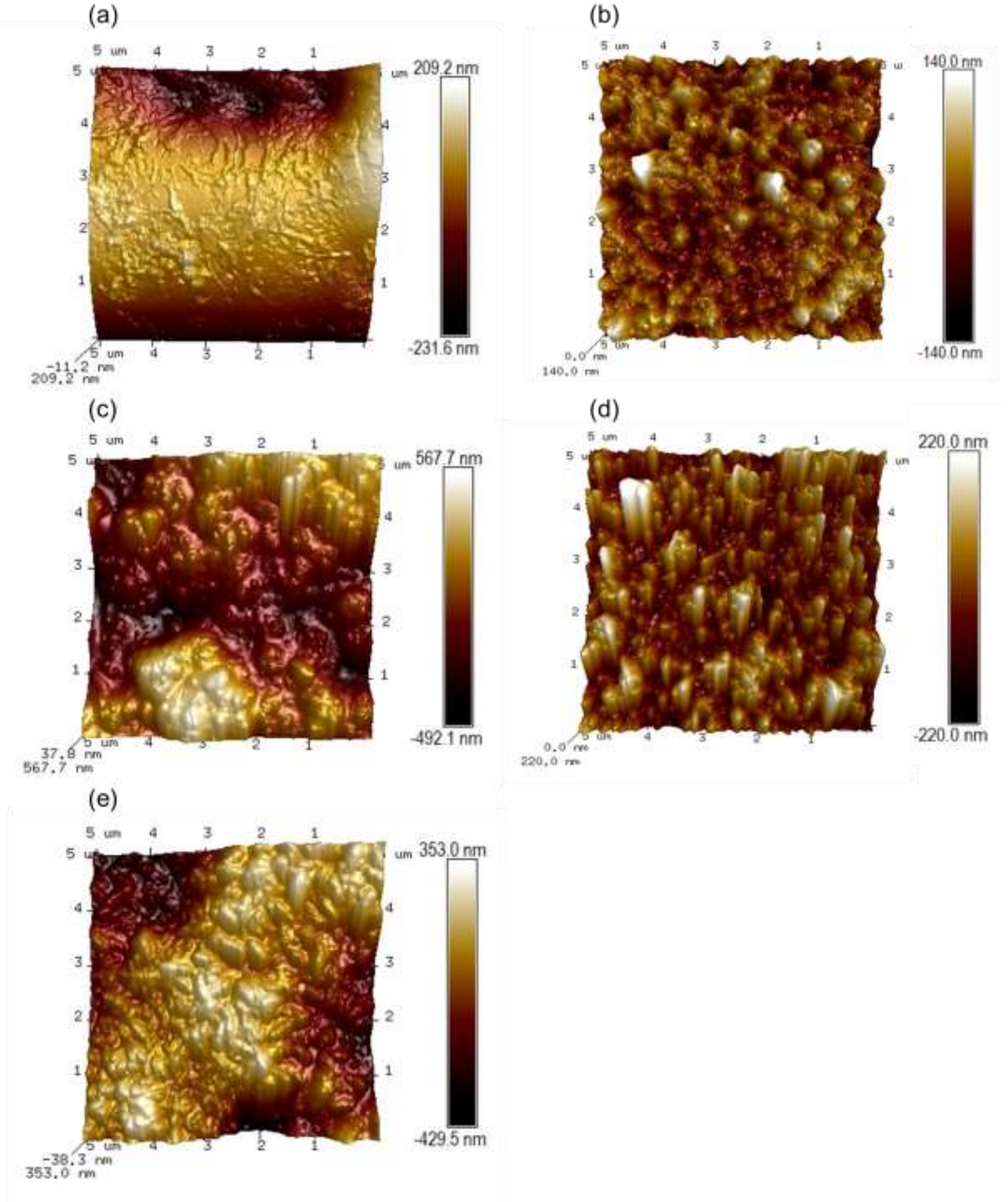

Figure S3: Atomic Force Microscopy profile for the electrochemically deposited thin film of synaptic active layer material of (a) SnSe (b) $SnSe_{0.75}Te_{0.25}$ (c) $SnSe_{0.5}Te_{0.5}$ (d) $SnSe_{0.25}Te_{0.75}$ (e) SnTe.

Figure S4:

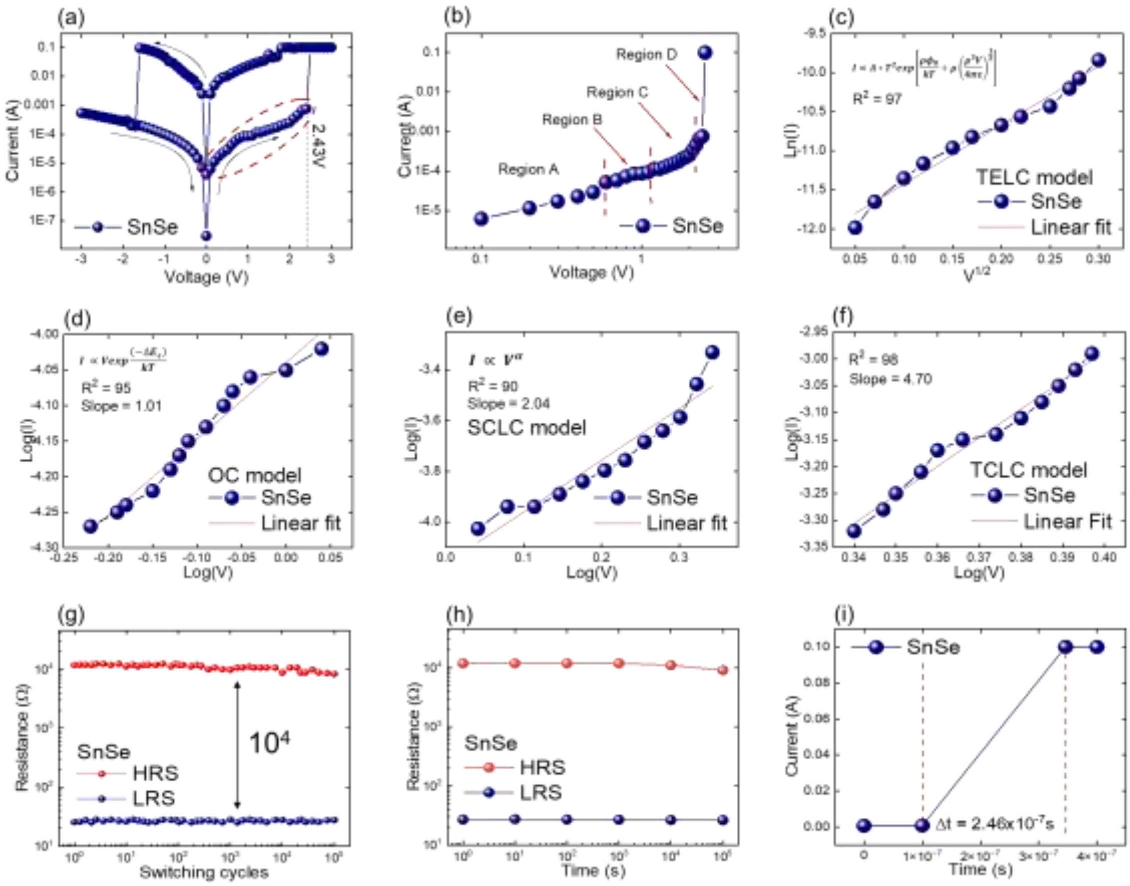

Figure S4: Synaptic switching mechanism and characteristics of neuristor with SnSe as active layer (a) Current-Voltage characteristics demonstrating the neuristors switching from high resistance state to low resistance state at a higher threshold voltage of ~2.43V (b) shows the different switching regions/stages in the synaptic switching mechanism (c) initial synaptic switching stage induced by thermionic emission of charges into the neuristors active layer (d) ohmic conduction region (e) synaptic switching current induced by the accumulation of charges in the active layer following space charge limited current (f) trapped charge limited current induced switching stage leading to the change in resistance state of the neuristor (g) measure of endurance and on/off ratio for the synaptic switching stability (h) shows the charge retention stability in the SnSe neuristors with respect to time (i) shows the switching time for the transition from high resistance state to low resistance state 0.246 µs.

Figure S5

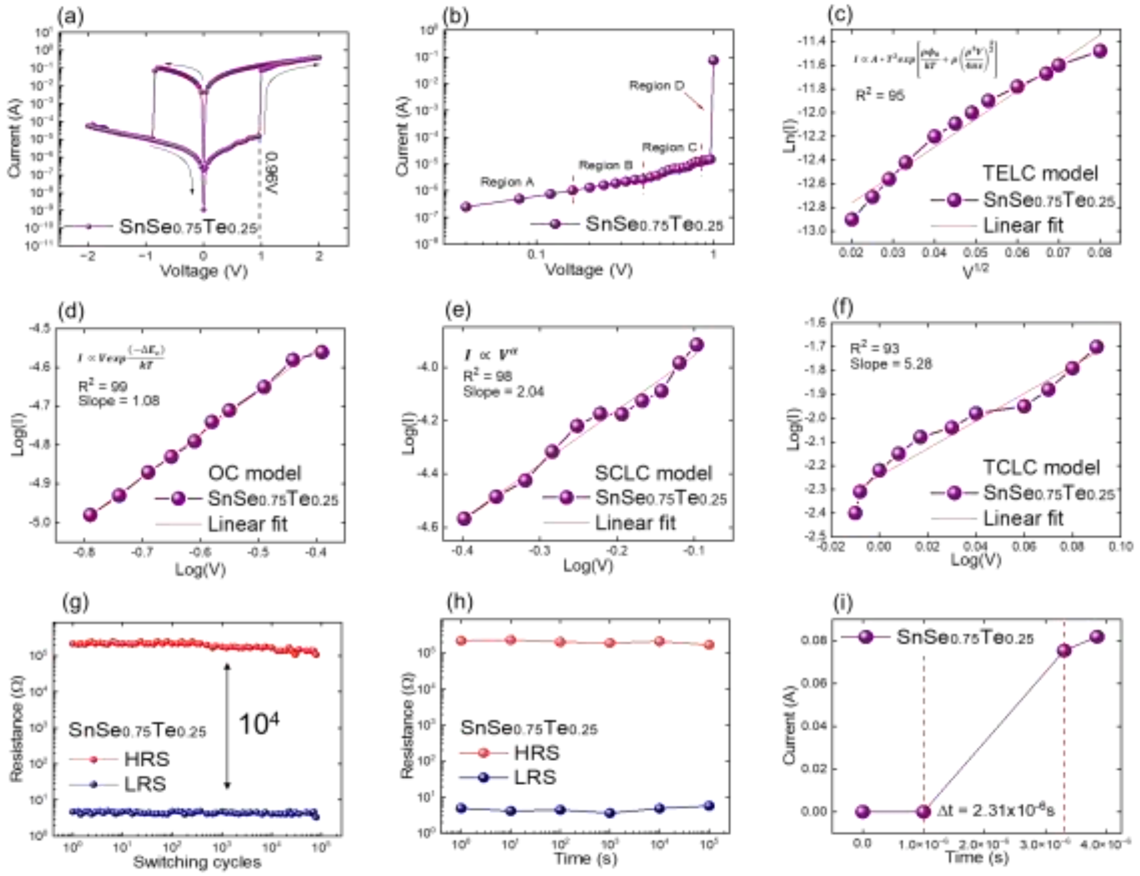

Figure S5: Synaptic switching mechanism and characteristics of neuristors with $SnSe_{0.75}Te_{0.25}$ as active layer (a) Current-Voltage characteristics demonstrating the neuristors switching from high resistance state to low resistance state at a lower threshold voltage of ~0.96V (b) shows the different switching regions/stages in the synaptic switching mechanism (c) initial synaptic switching stage induced by thermionic emission of charges into the neuristors active layer (d) ohmic conduction region (e) synaptic switching current induced by the accumulation of charges in the active layer following space charge limited current (f) trapped charge limited current induced switching stage leading to the change in resistance state of the neuristor (g) measure of endurance and on/off ratio for the synaptic switching stability (h) shows the charge retention stability in the $SnSe_{0.75}Te_{0.25}$ neuristors with respect to time (i) shows the switching time for the transition from high resistance state to low resistance state in 2.31 μs.

Figure S6

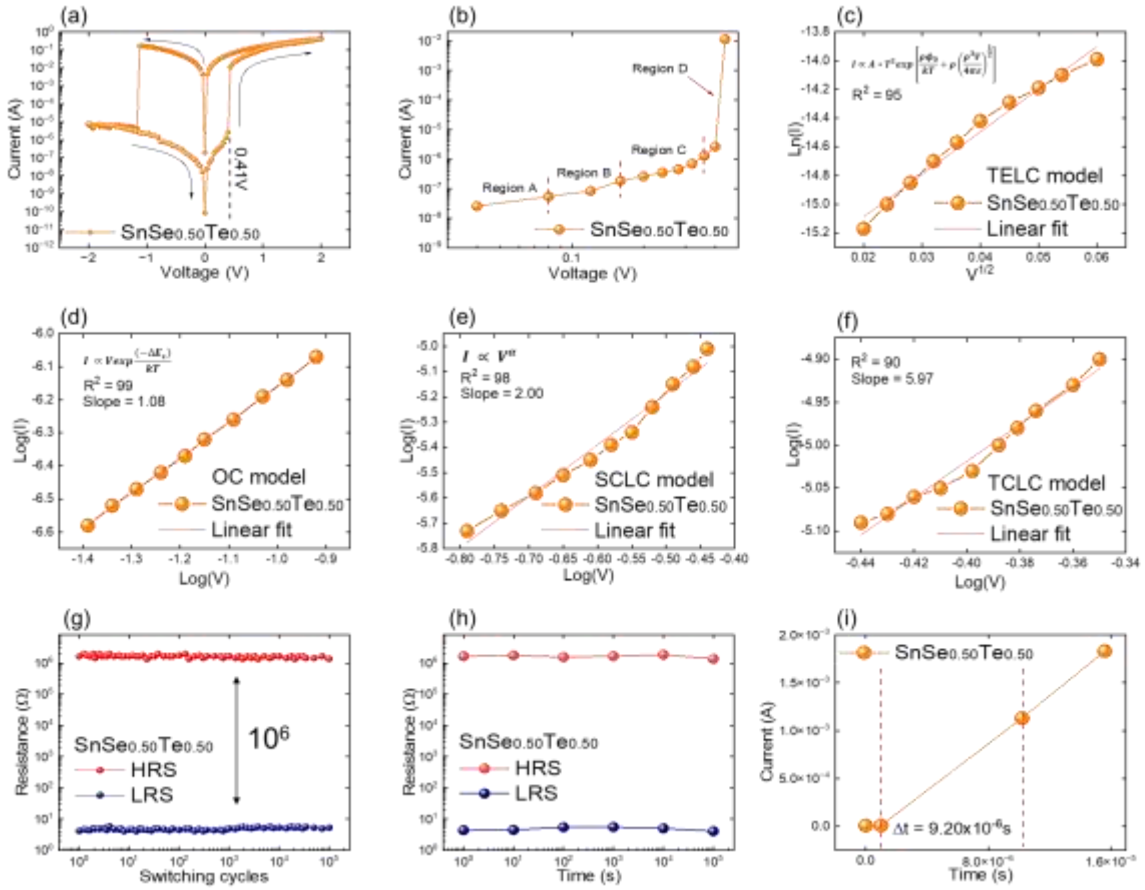

Figure S6: Synaptic switching mechanism and characteristics of neuristors with $SnSe_{0.5}Te_{0.5}$ as active layer (a) Current-Voltage characteristics demonstrating the neuristors switching from high resistance state to low resistance state at a lowest threshold voltage of ~0.41V (b) shows the different switching regions/stages in the synaptic switching mechanism (c) initial synaptic switching stage induced by thermionic emission of charges into the neuristors active layer (d) ohmic conduction region (e) synaptic switching current induced by the accumulation of charges in the active layer following space charge limited current (f) trapped charge limited current induced switching stage leading to the change in resistance state of the neuristor (g) measure of endurance and on/off ratio for the synaptic switching stability (h) shows the charge retention stability in the $SnSe_{0.5}Te_{0.5}$ neuristors with respect to time (i) shows the switching time for the transition from high resistance state to low resistance state in 9.2 μs.

Figure S7

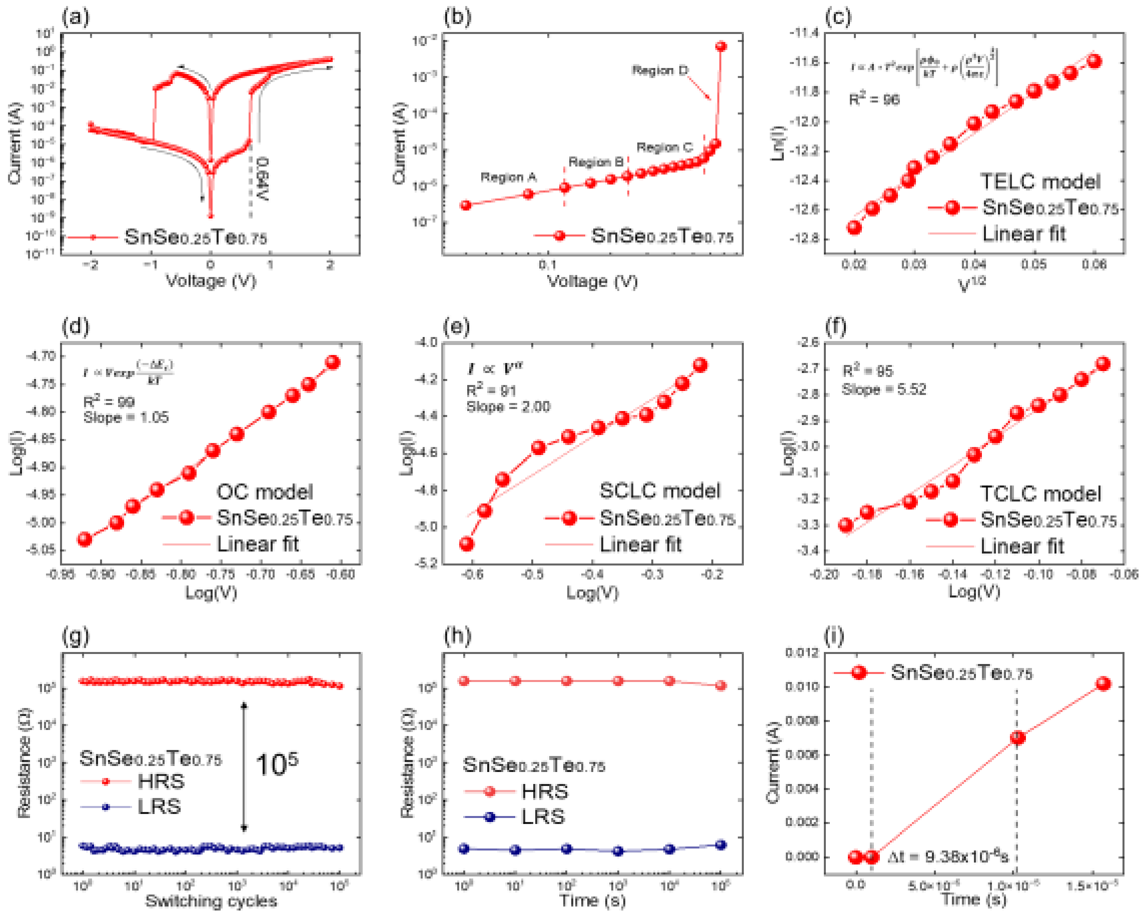

Figure S7: Synaptic switching mechanism and characteristics of neuristors with $SnSe_{0.25}Te_{0.75}$ as active layer (a) Current-Voltage characteristics demonstrating the neuristors switching from high resistance state to low resistance state at a lower threshold voltage of ~0.64V (b) shows the different switching regions/stages in the synaptic switching mechanism (c) initial synaptic switching stage induced by thermionic emission of charges into the neuristors active layer (d) ohmic conduction region (e) synaptic switching current induced by the accumulation of charges in the active layer following space charge limited current (f) trapped charge limited current induced switching stage leading to the change in resistance state of the neuristor (g) measure of endurance and on/off ratio for the synaptic switching stability (h) shows the charge retention stability in the $SnSe_{0.25}Te_{0.75}$ neuristors with respect to time (i) shows the switching time for the transition from high resistance state to low resistance state in 9.38 µs.

Figure S8

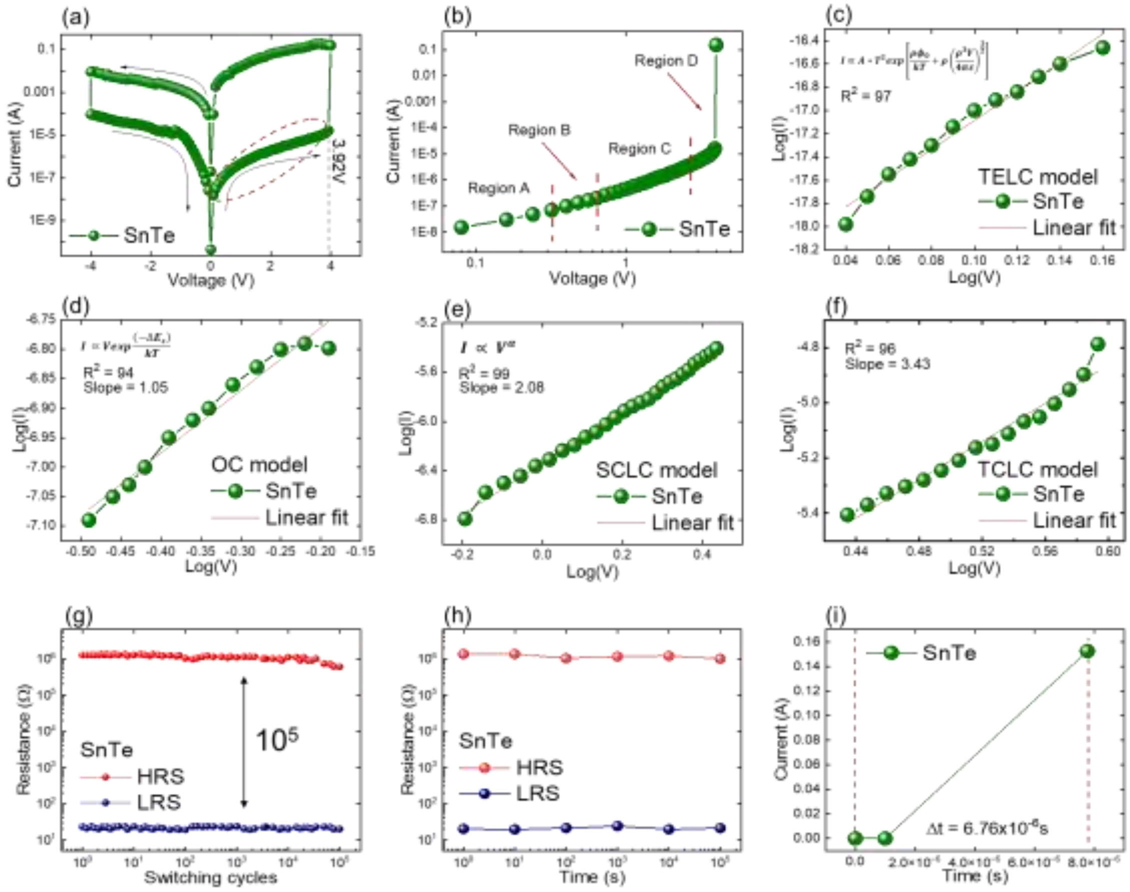

Figure S8: Synaptic switching mechanism and characteristics of neuristors with SnTe as active layer (a) Current-Voltage characteristics demonstrating the neuristors switching from high resistance state to low resistance state at a high threshold voltage of ~3.92V (b) shows the different switching regions/stages in the synaptic switching mechanism (c) initial synaptic switching stage induced by thermionic emission of charges into the neuristors active layer (d) ohmic conduction region (e) synaptic switching current induced by the accumulation of charges in the active layer following space charge limited current (f) trapped charge limited current induced switching stage leading to the change in resistance state of the neuristor (g) measure of endurance and on/off ratio for the synaptic switching stability (h) shows the charge retention stability in the SnTe neuristors with respect to time (i) shows the switching time for the transition from high resistance state to low resistance state in 6.76 µs.

Figure S9

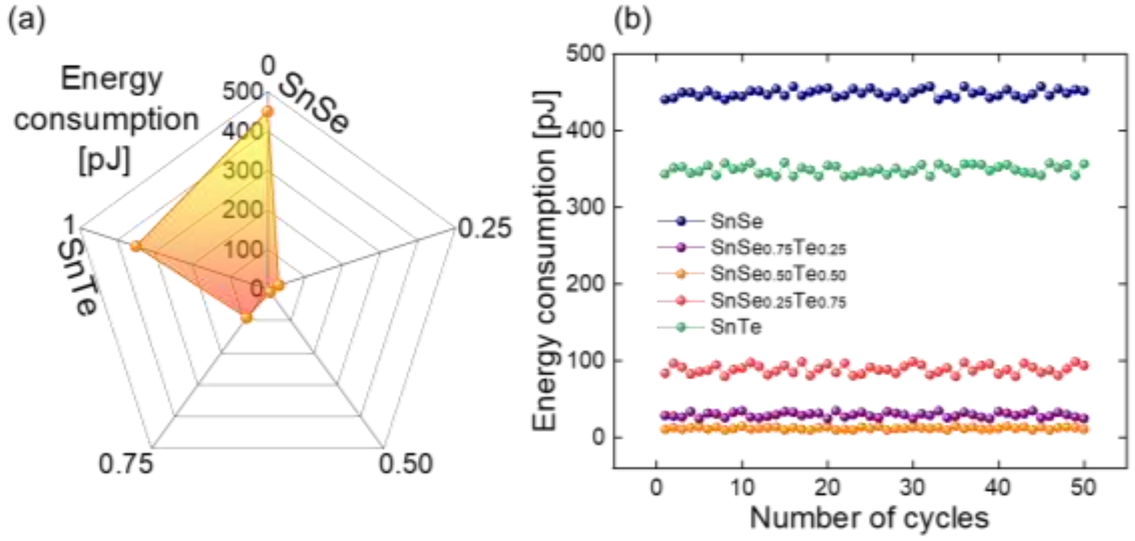

Figure S9: Energy Consumption for synaptic switching in our quantum topological neuristors (a) Comparison of standardized energy consumption for varied proportions of with $SnSe_{1-x}Te_x$ (x= 0, 0.25, 0.5, 0.75, 1) (b) Data driven measurement set for reliability in energy consumption for $SnSe_{1-x}Te_x$ (x= 0, 0.25, 0.5, 0.75, 1).

Figure S10

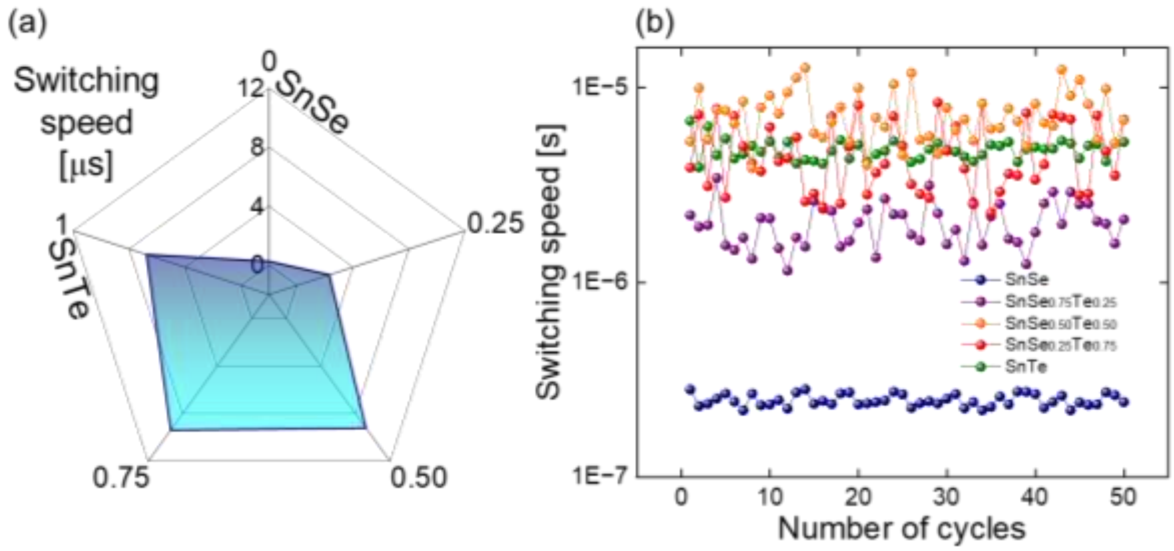

Figure S10: Switching speed of synaptic switching in our quantum topological neuristors (a) Comparison of standardized switching speed for varied proportions of $SnSe_{1-x}Te_x$ (x= 0, 0.25, 0.5, 0.75, 1) (b) Data driven measurement set for reliability in switching speed for $SnSe_{1-x}Te_x$ (x= 0, 0.25, 0.5, 0.75, 1).

Figure S11

Switching energy comparison between different group of active materials [1-42]

Here we present the comparison of energy required to perform the switching of the device state from high resistance(off) state to the low resistance(on) state among different group of materials including oxides, inorganic compounds and organic materials. Our comparison of energy consumption illustrates the properties of the materials to perform the switching process. Here oxide materials are show a mean average energy consumption range of 10^{-6} J, inorganic materials via their narrow bandgap nature required lower energy ~10^{-7} J to change their switching states. On the other hand, organic materials require ionic conduction mechanism to induce the switching process hence it utilizes high energy 10^{-3} J for switching process. Hence to overcome this issue of high energy requirements for switching, we introduce the use of topological insulator materials which performs an energy efficient switching process via edge state conduction mechanism. As shown in the figure S11, the energy requirement for the switching process in < 10 pJ.

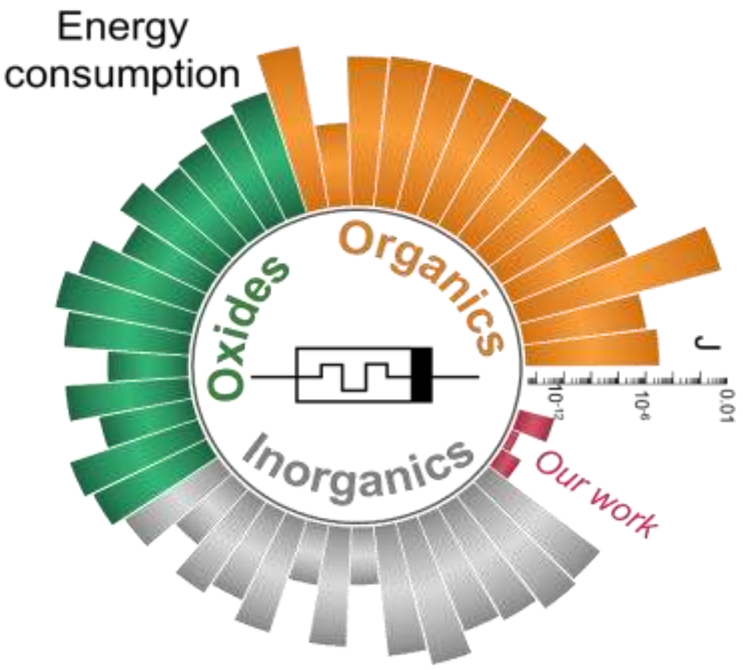

Figure S11: Comparison of energy consumption in organics, inorganics and oxides based switching devices with topological insulator based neuristors.

Table S1: Comparison of energy consumption in organics, inorganics and oxides-based switching devices with topological insulator based neuristors.

No.	Active Material	Energy consumption [J]	Reference
**Organic-based devices**			
1	SHP, PDMS, MPU, IU, AgFs	$2x10^{-3}$	[1]
2	$CsPbBr_3$	$9x10^{-4}$	[2]

3	$PrPyr[PbI_3]$	$8x10^{-1}$	[3]
4	Silk-Ag nanowaires	$11x10^{-4}$	[4]
5	Polydopamine	$14x10^{-3}$	[5]
6	Poly(N-vinylcarbzaole)	$14x10^{-3}$	[6]
7	PI:PCBM	$3.8x10^{-3}$	[7]
8	$MAPbI_{3-x}Cl_x$	$10x10^{-3}$	[8]
9	$CH_3NH_3PbI_{3-x}Cl_x$	$12x10^{-3}$	[9]
10	MIL-53/GaInSn@PDMS	$13x10^{-3}$	[10]
11	Ni-BTA	$14x10^{-3}$	[11]
12	X55, PCBM	$12x10^{-3}$	[12]
13	Su8	$50x10^{-6}$	[13]
14	BMThCE	$42x10^{-3}$	[14]
**Oxide-based devices**			
15	TiO_2/Au NP	$1.9x10^{-3}$	[15]
16	HfO_x	$900x10^{-6}$	[16]
17	$TaOx/HfAl_yO_x$	$300x10^{-6}$	[17]
18	TaO_x	$2.2x10^{-4}$	[18]
19	Hf_6O	$6.5x10^{-4}$	[19]
20	Al_2O_3/TiO_{2-x}	$100x10^{-6}$	[20]
21	Al_2O_3	$1.1x10^{-3}$	[21]
22	TiO_2	$3.1x10^{-3}$	[22]
23	$HfO_x/TiO_x/HfO_x/TiO_x$	$1x10^{-3}$	[23]
24	GeO_x/HfON	$3x10^{-5}$	[24]
25	HfO_x	$900x10^{-6}$	[25]
26	$TaOy/Ta_2O_{5-x}$	$9x10^{-5}$	[26]
27	TiO_x	$2.3x10^{-3}$	[27]
28	Y_2O_3	$1.2x10^{-3}$	[28]
**Inorganic-based devices**			
29	SiGe	$4x10^{-4}$	[29]
30	Cu_2S	$2.2x10^{-5}$	[30]
31	CdS	$4x10^{-4}$	[31]
32	Ag_2Se	$1.6x10^{-4}$	[32]
33	GeTe	$9x10^{-4}$	[33]
34	BN	$8x10^{-5}$	[34]
35	$Ge_2Sb_2Te_5$	$1x10^{-3}$	[35]
36	CNT	$9x10^{-6}$	[36]
37	Bi_2Se_3-PMMA	$2.5x10^{-3}$	[37]
38	Bi_2Te_3	$9x10^{-3}$	[38]
39	MoS_2	$1.4x10^{-3}$	[39]
40	$MoTe_2$	$2.3x10^{-3}$	[40]
41	CNT	$6x10^{-3}$	[41]
42	$CsPbBr_3$ QDs	$4x10^{-3}$	[42]
**Quantuum Topological Insulators-based devices (Our work)**			
**44**	**SnSe**	$\mathbf{45x10^{-11}}$	**this work**
**45**	$\mathbf{SnSe_{0.75}Te_{0.25}}$	$\mathbf{30x10^{-12}}$	**this work**
**46**	$\mathbf{SnSe_{0.50}Te_{0.50}}$	$\mathbf{10x10^{-12}}$	**this work**
**47**	$\mathbf{SnSe_{0.25}Te_{0.75}}$	$\mathbf{90x10^{-12}}$	**this work**
**48**	**SnTe**	$\mathbf{35x10^{-11}}$	**this work**

Figure S12: Switching speed comparison between different group of active materials [43-66]

Here we present the comparison of transition time required to perform the switching of the device state from high resistance(off) state to the low resistance(on) state among different group of materials including oxides, inorganic compounds and organic materials. Our comparison of transition time illustrates the capability of the materials to perform the switching process. Here oxide materials are showing a mean average switching speed range of 10^{-4} s, inorganic materials via their good electronic mobility nature required 10^{-4} to 10^{-5} s to change their switching states. On the other hand, organic materials require filament formation mechanism to induce the switching process hence it utilizes higher time of 10^{-3} s for switching process. By using of topological insulator materials, the decoupling between the energy consumption and the switching speed can be achieved, the presence of topological edge state transports the charges faster leading to achieve switching speed < 10 μs as shown in the figure S12.

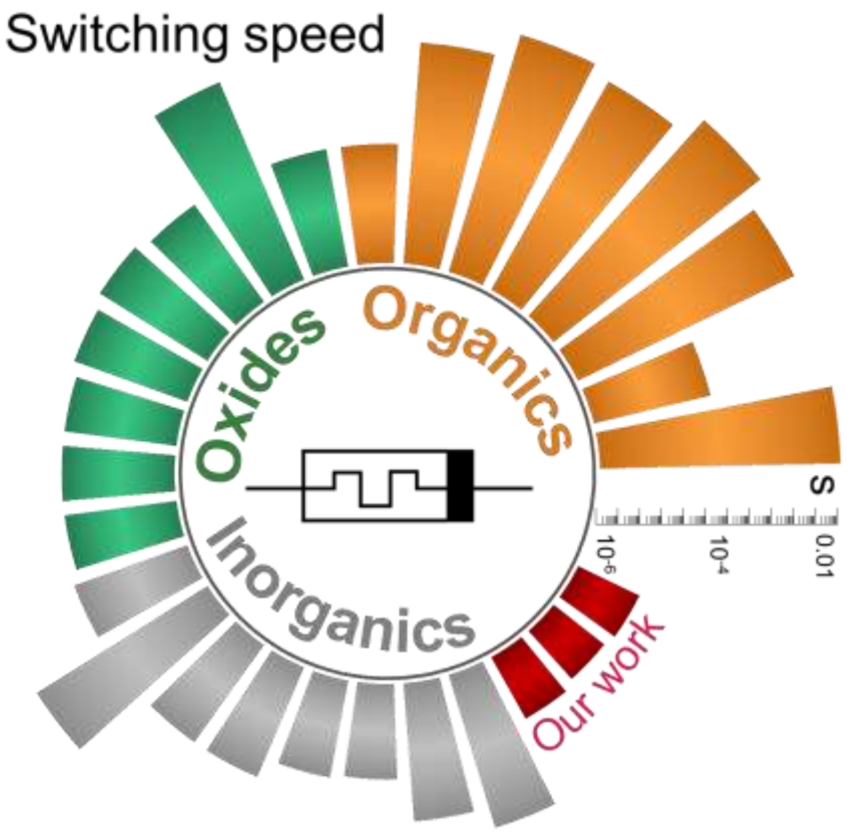

Figure S12: Comparison of switching speed in organics, inorganics and oxides-based switching devices with topological insulator based neuristors.

Table S2: Comparison of switching speed in organics, inorganics and oxides-based switching devices with topological insulator based neuristors.

No.	Active Material	Switching speed [s]	Reference
**Organic-based devices**			
1	P(VDF–TrFE)/MoS_2	10^{-2}	[43]
2	APP	10^{-7}	[44]
3	PVPy:Au@Ag NPs	10^{-2}	[45]

4	Lignin	0.1	[46]
5	PEDOT:PSS/Nafion	10^{-3}	[47]
6	PEDOT:PSS/TFSI(PVDF-HFP)	$25x10^{-3}$	[48]
7	$RbAg_4I_3$	$2x10^{-3}$	[49]
8	PI:PCBM	10^{-7}	[50]
Oxide-based devices			
9	SiO_xN_y:Ag	$2x10^{-4}$	[51]
10	MnO_3	10^{-3}	[52]
11	SiON:Ag	10^{-4}	[53]
12	$LaAlO_3/SrTiO_3$:Nb	$0.25x10^{-3}$	[54]
13	$CoO/SrTiO_3$:Nb	$2x10^{-3}$	[55]
14	α-MoO_3	$1x10^{-3}$	[56]
15	$NiAlOx/Al_2O_{3-x}$	$5x10^{-2}$	[57]
16	Al_2O_3	$0.8x10^{-3}$	[58]
**Inorganic-based devices**			
17	$MAPbBr_3$/BCCP	$7x10^{-5}$	[59]
18	$LaAlO_3/SrTiO_3$:Nb	$2x10^{-2}$	[60]
19	$PMMA/CsPbI_3$	$64x10^{-5}$	[61]
20	Cu_2S	10^{-4}	[62]
21	MoS_2	10^{-3}	[63]
22	$MAPbClBr_2$	$25x10^{-3}$	[64]
23	$MAPbI_3$	$6.4x10^{-4}$	[65]
24	SiGe	$5x10^{-6}$	[66]
**Quantum Topological Insulators-based devices (Our work)**			
**25**	**SnSe**	**$2.46x10^{-7}$**	**this work**
**26**	**$SnSe_{0.75}Te_{0.25}$**	**$2.31x10^{-6}$**	**this work**
**27**	**$SnSe_{0.50}Te_{0.50}$**	**$9.20x10^{-6}$**	**this work**
**28**	**$SnSe_{0.25}Te_{0.75}$**	**$9.38x10^{-6}$**	**this work**
**29**	**SnTe**	**$6.76x10^{-6}$**	**this work**

Table S3: Capacitance change between presynaptic neuron and postsynaptic neuron.

Threshold Voltage (V)	Capacitance change (nF)
0	0.01584
0.1	0.04729
0.2	0.618
0.3	0.8658
0.4	1

References

[1] J. Park, D. Seong, Y. J. Park, S. H. Park, H. Jung, Y. Kim, H. W. Baac, M. Shin, S. Lee, M. Lee, D. Son, *Nat Commun* **2022**, *13*, 5233.
[2] M. C. Yen, C. J. Lee, K. H. Liu, Y. Peng, J. Leng, T. H. Chang, C. C. Chang, K. Tamada, Y. J. Lee, *Nat Commun* **2021**, *12*, 4460.
[3] R. A. John, N. Shah, S. K. Vishwanath, S. E. Ng, B. Febriansyah, M. Jagadeeswararao, C. H. Chang, A. Basu, N. Mathews, *Nat Commun* **2021**, *12*, 3681.
[4] W. Wang, M. Wang, E. Ambrosi, A. Bricalli, M. Laudato, Z. Sun, X. Chen, D. Ielmini, *Nat Commun* **2019**, *10*, 81.
[5] H. Bae, D. Kim, M. Seo, I. K. Jin, S. B. Jeon, H. M. Lee, S. H. Jung, B. C. Jang, G. Son, K. Yu, S. Y. Choi, Y. K. Choi, *Adv Mater Technol* **2019**, *4*, 1900151.
[6] H. F. Ling, M. D. Yi, M. Nagai, L. H. Xie, L. Y. Wang, B. Hu, W. Huang, *Advanced Materials* **2017**, *29*, 1701333.
[7] S. T. Han, Y. Zhou, V. A. L. Roy, *Advanced Materials* **2013**, *25*, 5425.
[8] J. Choi, J. S. Han, K. Hong, S. Y. Kim, H. W. Jang, *Advanced Materials* **2018**, *30*, 1704002.
[9] E. J. Yoo, M. Lyu, J. H. Yun, C. J. Kang, Y. J. Choi, L. Wang, *Advanced Materials* **2015**, *27*, 6170.
[10] X. Yi, Z. Yu, X. Niu, J. Shang, G. Mao, T. Yin, H. Yang, W. Xue, P. Dhanapal, S. Qu, G. Liu, R. W. Li, *Adv Electron Mater* **2019**, *5*, 1800655.
[11] X. F. Cheng, E. B. Shi, X. Hou, J. Shu, J. H. He, H. Li, Q. F. Xu, N. J. Li, D. Y. Chen, J. M. Lu, *Adv Electron Mater* **2017**, *3*, 1700107.
[12] Y. Y. Zhao, W. J. Sun, M. G. Wang, J. H. He, J. M. Lu, *Adv Mater Technol* **2020**, *5*, 1900681.
[13] A. Subramanian, N. Tiwale, K. Kisslinger, C. Y. Nam, *Adv Electron Mater* **2022**, *8*, 2200172.
[14] H. Ling, K. Tan, Q. Fang, X. Xu, H. Chen, W. Li, Y. Liu, L. Wang, M. Yi, R. Huang, Y. Qian, L. Xie, W. Huang, *Adv Electron Mater* **2017**, *3*, 1600416.
[15] D. Son, J. Lee, S. Qiao, R. Ghaffari, J. Kim, J. E. Lee, C. Song, S. J. Kim, D. J. Lee, S. W. Jun, S. Yang, M. Park, J. Shin, K. Do, M. Lee, K. Kang, C. S. Hwang, N. Lu, T. Hyeon, D. H. Kim, *Nat Nanotechnol* **2014**, *9*, 397.
[16] M. Wang, C. Bi, L. Li, S. Long, Q. Liu, H. Lv, N. Lu, P. Sun, M. Liu, *Nat Commun* **2014**, *5*, 4598.
[17] P. Yao, H. Wu, B. Gao, S. B. Eryilmaz, X. Huang, W. Zhang, Q. Zhang, N. Deng, L. Shi, H. S. P. Wong, H. Qian, *Nat Commun* **2017**, *8*, 15199.
[18] M. J. Lee, C. B. Lee, D. Lee, S. R. Lee, M. Chang, J. H. Hur, Y. B. Kim, C. J. Kim, D. H. Seo, S. Seo, U. I. Chung, I. K. Yoo, K. Kim, *Nat Mater* **2011**, *10*, 625.
[19] Y. Zhang, G. Q. Mao, X. Zhao, Y. Li, M. Zhang, Z. Wu, W. Wu, H. Sun, Y. Guo, L. Wang, X. Zhang, Q. Liu, H. Lv, K. H. Xue, G. Xu, X. Miao, S. Long, M. Liu, *Nat Commun* **2021**, *12*, 7232.
[20] H. Kim, M. R. Mahmoodi, H. Nili, D. B. Strukov, *Nat Commun* **2021**, *12*, 5198.
[21] K. Y. Shin, Y. Kim, F. v. Antolinez, J. S. Ha, S. S. Lee, J. H. Park, *Adv Electron Mater* **2016**, *2*, 1600233.
[22] S. H. Kim, G. W. Baek, J. Yoon, S. Seo, J. Park, D. Hahm, J. H. Chang, D. Seong, H. Seo, S. Oh, K. Kim, H. Jung, Y. Oh, H. W. Baac, B. Alimkhanuly, W. K. Bae, S. Lee, M. Lee, J. Kwak, J. H. Park, D. Son, *Advanced Materials* **2021**, *33*, 2104690.
[23] S. Yu, B. Gao, Z. Fang, H. Yu, J. Kang, H. S. P. Wong, *Advanced Materials* **2013**, *25*, 1774.
[24] C. H. Cheng, F. S. Yeh, A. Chin, *Advanced Materials* **2011**, *23*, 902.
[25] C. Wang, H. Wu, B. Gao, W. Wu, L. Dai, X. Li, H. Qian, *Adv Electron Mater* **2017**, *3*, 1700263.
[26] Y. Yang, J. Lee, S. Lee, C. H. Liu, Z. Zhong, W. Lu, *Advanced Materials* **2014**, *26*, 3693.
[27] R. Hu, X. Li, J. Tang, Y. Li, X. Zheng, B. Gao, H. Qian, H. Wu, *Adv Electron Mater* **2022**, *8*, 2100827.
[28] S. Petzold, E. Piros, R. Eilhardt, A. Zintler, T. Vogel, N. Kaiser, A. Radetinac, P. Komissinskiy, E. Jalaguier, E. Nolot, C. Charpin-Nicolle, C. Wenger, L. Molina-Luna, E. Miranda, L. Alff, *Adv Electron Mater* **2020**, *6*, 2000439.
[29] S. Choi, S. H. Tan, Z. Li, Y. Kim, C. Choi, P. Y. Chen, H. Yeon, S. Yu, J. Kim, *Nat Mater* **2018**, *17*, 335.
[30] P. H. Liu, C. C. Lin, A. Manekkathodi, L. J. Chen, *Nano Energy* **2015**, *15*, 362.
[31] Y. C. Ju, S. Kim, T. G. Seong, S. Nahm, H. Chung, K. Hong, W. Kim, *Small* **2012**, *8*, 2849.
[32] J. Jang, F. Pan, K. Braam, V. Subramanian, *Advanced Materials* **2012**, *24*, 3573.
[33] S. J. Choi, G. S. Park, K. H. Kim, S. Cho, W. Y. Yang, X. S. Li, J. H. Moon, K. J. Lee, K. Kim, *Advanced Materials* **2011**, *23*, 3272.
[34] G. U. Siddiqui, M. M. Rehman, Y. J. Yang, K. H. Choi, *J Mater Chem C Mater* **2017**, *5*, 862.
[35] D. Deleruyelle, M. Putero, T. Ouled-Khachroum, M. Bocquet, M. v. Coulet, X. Boddaert, C. Calmes, C. Muller, *Solid State Electron* **2013**, *79*, 159.
[36] C. L. Tsai, F. Xiong, E. Pop, M. Shim, *ACS Nano* **2013**, *7*, 5360.

[37] B. Das, P. K. Sarkar, N. S. Das, S. Sarkar, K. K. Chattopadhyay, *J Appl Phys* **2018**, *124*, 124503.
[38] Y. Bao, Z. Ren, H. Li, K. Huang, *J Phys D Appl Phys* **2019**, *52*, 075103.
[39] B. Tang, H. Veluri, Y. Li, Z. G. Yu, M. Waqar, J. F. Leong, M. Sivan, E. Zamburg, Y. W. Zhang, J. Wang, A. V. Y. Thean, *Nat Commun* **2022**, *13*, 3037.
[40] F. Zhang, H. Zhang, S. Krylyuk, C. A. Milligan, Y. Zhu, D. Y. Zemlyanov, L. A. Bendersky, B. P. Burton, A. v. Davydov, J. Appenzeller, *Nat Mater* **2019**, *18*, 55.
[41] A. D. Liao, P. T. Araujo, R. Xu, M. S. Dresselhaus, *Nat Commun* **2014**, *5*, 5673.
[42] Y. Wang, Z. Lv, Q. Liao, H. Shan, J. Chen, Y. Zhou, L. Zhou, X. Chen, V. A. L. Roy, Z. Wang, Z. Xu, Y. J. Zeng, S. T. Han, *Advanced Materials* **2018**, *30*, 1800327.
[43] S. Kim, K. Heo, S. Lee, S. Seo, H. Kim, J. Cho, H. Lee, K. B. Lee, J. H. Park, *Nanoscale Horiz* **2021**, *6*, 139.
[44] Y. Y. Zhao, W. J. Sun, J. Wang, J. H. He, H. Li, Q. F. Xu, N. J. Li, D. Y. Chen, J. M. Lu, *Adv Funct Mater* **2020**, *30*, 2004245.
[45] L. Zhou, J. Y. Mao, Y. Ren, J. Q. Yang, S. R. Zhang, Y. Zhou, Q. Liao, Y. J. Zeng, H. Shan, Z. Xu, J. Fu, Y. Wang, X. Chen, Z. Lv, S. T. Han, V. A. L. Roy, *Small* **2018**, *14*, 1800288.
[46] Y. Park, J. S. Lee, *ACS Nano* **2017**, *11*, 8962.
[47] Y. van de Burgt, E. Lubberman, E. J. Fuller, S. T. Keene, G. C. Faria, S. Agarwal, M. J. Marinella, A. Alec Talin, A. Salleo, *Nat Mater* **2017**, *16*, 414.
[48] Y. Li, T. P. Xiao, C. H. Bennett, E. Isele, A. Melianas, H. Tao, M. J. Marinella, A. Salleo, E. J. Fuller, A. A. Talin, *Front Neurosci* **2021**, *15*, 636127.
[49] Q. Lai, L. Zhang, Z. Li, W. F. Stickle, R. S. Williams, Y. Chen, *Advanced Materials* **2010**, *22*, 2448.
[50] S. T. Han, Y. Zhou, V. A. L. Roy, *Advanced Materials* **2013**, *25*, 5425.
[51] Z. Wang, S. Joshi, S. E. Savel'ev, H. Jiang, R. Midya, P. Lin, M. Hu, N. Ge, J. P. Strachan, Z. Li, Q. Wu, M. Barnell, G. L. Li, H. L. Xin, R. S. Williams, Q. Xia, J. J. Yang, *Nat Mater* **2017**, *16*, 101.
[52] C. sen Yang, D. S. Shang, N. Liu, G. Shi, X. Shen, R. C. Yu, Y. Q. Li, Y. Sun, *Advanced Materials* **2017**, *29*, 1700906.
[53] Z. Wang, S. Joshi, S. E. Savel'ev, H. Jiang, R. Midya, P. Lin, M. Hu, N. Ge, J. P. Strachan, Z. Li, Q. Wu, M. Barnell, G. L. Li, H. L. Xin, R. S. Williams, Q. Xia, J. J. Yang, *Nat Mater* **2017**, *16*, 101.
[54] J. T. Jang, D. Ko, G. Ahn, H. R. Yu, H. Jung, Y. S. Kim, C. Yoon, S. Lee, B. H. Park, S. J. Choi, D. M. Kim, D. H. Kim, *Solid State Electron* **2018**, *140*, 139.
[55] L. Zhao, J. Xu, X. Shang, X. Li, Q. Li, S. Li, *R Soc Open Sci* **2019**, *6*, 181098.
[56] Yang, C. S., Shang, D. S., Liu, N., Shi, G., Shen, X., Yu, R. C., Li, Y. Q., Sun, Y., *Adv. Mater.* **2017**, 29, 1700906.
[57] Y. W. Dai, L. Chen, W. Yang, Q. Q. Sun, P. Zhou, P. F. Wang, S. J. Ding, D. W. Zhang, F. Xiao, *IEEE Electron Device Letters* **2014**, *35*, 915.
[58] B. Sarkar, B. Lee, V. Misra, *Semicond Sci Technol* **2015**, *30*, 105014.
[59] W. Xu, H. Cho, Y. H. Kim, Y. T. Kim, C. Wolf, C. G. Park, T. W. Lee, *Advanced Materials* **2016**, *28*, 5916.
[60] J. T. Jang, D. Ko, G. Ahn, H. R. Yu, H. Jung, Y. S. Kim, C. Yoon, S. Lee, B. H. Park, S. J. Choi, D. M. Kim, D. H. Kim, *Solid State Electron* **2018**, *140*, 139.
[61] J. Di, J. Du, Z. Lin, S. Liu, J. Ouyang, J. Chang, *InfoMat* **2021**, *3*, 293.
[62] F T. Sakamoto, H. Sunamura, H. Kawaura, T. Hasegawa, T. Nakayama, M. Aonob, *Appl Phys Lett* **2003**, *82*, 3032.
[63] S. Kim, K. Heo, S. Lee, S. Seo, H. Kim, J. Cho, H. Lee, K. B. Lee, J. H. Park, *Nanoscale Horiz* **2021**, *6*, 139.
[64] H. Yu, J. Gong, H. Wei, W. Huang, W. Xu, *Mater Chem Front* **2019**, *3*, 941.
[65] B. Li, W. Hui, X. Ran, Y. Xia, F. Xia, L. Chao, Y. Chen, W. Huang, *J Mater Chem C Mater* **2019**, *7*, 7476.
[66] S. Choi, S. H. Tan, Z. Li, Y. Kim, C. Choi, P. Y. Chen, H. Yeon, S. Yu, J. Kim, *Nat Mater* **2018**, *17*, 335.
